# Safety and efficacy of new staple-line reinforcement in lung resection: a prospective study of 48 patients

**DOI:** 10.1007/s00595-024-02798-x

**Published:** 2024-02-21

**Authors:** Suguru Mitsui, Yugo Tanaka, Megumi Nishikubo, Takefumi Doi, Shinya Tane, Daisuke Hokka, Yuji Mitomo, Yoshimasa Maniwa

**Affiliations:** 1 Division of Thoracic Surgery, Kobe University Graduate School of Medicine, 7-5-2 Kusunoki-cho, Chuou-ku, Kobe, Hyogo 650-0017 Japan; 2 Clinical and Translational Research Center, Kobe University Hospital, Kobe, Hyogo Japan

**Keywords:** Bioabsorbable staple-line reinforcement, Pulmonary resection, Air leakage, Automated suturing device

## Abstract

**Purpose:**

To evaluate the safety and efficacy of new staple-line reinforcement (SLR) in pulmonary resection through a prospective study and to compare the results of this study with historical control data in an exploratory study.

**Methods:**

The subjects of this study were 48 patients who underwent thoracoscopic lobectomy. The primary endpoint was air leakage from the staple line. The secondary endpoints were the location of air leakage, duration of air leakage, and postoperative pulmonary complications.

**Results:**

The incidence of intraoperative air leakage from the staple line was 6.3%. Three patients had prolonged air leakage as a postoperative pulmonary complication. No malfunction was found in patients who underwent SLR with the stapling device. When compared with the historical group, the SLR group had a significantly lower incidence of air leakage from the staple line (6.3% vs. 28.5%, *P* < 0.001) and significantly shorter indwelling chest drainage time (*P* = 0.049) and length of hospital stay (*P* < 0.001).

**Conclusions:**

The use of SLR in pulmonary resection was safe and effective. When compared with conventional products, SLR could control intraoperative air leakage from the staple line and shorten time needed for indwelling chest drainage and the length of hospital stay.

**Supplementary Information:**

The online version contains supplementary material available at 10.1007/s00595-024-02798-x.

## Introduction

The treatment strategies for lung cancer are based on a comprehensive consideration of the histological type, progression, and patient background, but surgical therapy remains the mainstay of lung cancer treatment. Advances in surgical technology including minimally invasive thoracic surgery, such as video-assisted thoracic surgery (VATS) and robotic assisted thoracoscopic surgery (RATS) have improved postoperative recovery and the incidence of postoperative complications [1, 2]. The incidence of air leakage after pulmonary resection, one of the most common postoperative complications, has decreased with the use of advanced automated suturing devices and tissue reinforcement material, but it remains high [3]. Patients who undergo pulmonary resection require the installation of a chest drainage tube until the air leakage resolves; therefore, prolonged air leakage extends the length of hospital stay and escalates hospitalization costs [4, 5]. Conversely, early removal of the chest drainage tube promotes faster recovery by reducing pain and improving the patients’ respiratory function [6]. Therefore, the prevention of postoperative air leakage enables timely removal of chest drainage tubes and early rehabilitation; thereby, decreasing medical costs.

After pulmonary resection, air leakage may be detected in the perivascular, peribronchial, and staple-line areas, and is more frequent in patients with fragile lung parenchyma, such as those with chronic obstructive pulmonary disease (COPD) and interstitial pneumonia. The prevention of air leakage from staple lines is likely to depend on device performance; thus, improved devices could contribute to better postoperative outcomes. Recently, a new staple-line reinforcement (SLR) device made from synthetic absorbable materials used in polyglactin 910 and polydioxanone has become available, and is expected to prevent or improve postoperative air leakage. However, the safety and efficacy of this material in pulmonary resection have not been established. We conducted the current study to evaluate the safety and efficacy of SLR in pulmonary resection through a prospective study and to compare the results with historical control data in an exploratory study.

## Methods

The protocol of this single-center, single-arm prospective study was established in accordance with the principles stipulated in the Declaration of Helsinki and the research was approved by the Clinical Research Area Ethics Committee of Kobe University Graduate School of Medicine (#C210021). This clinical study is registered in the Japan registry of clinical trials as jRCT1052220008. All study cases were collected from May to December 2022.

This study included patients aged > 20 years who were scheduled to undergo lobectomy under VATS or RATS for suspected lung malignancies. The exclusion criteria were as follows: patients who had undergone previous ipsilateral pulmonary resection, patients with complete interlobar fissures of the lung, and patients with pulmonary resection larger than one lobe.

Patient information was extracted by physical examination, medical history taking, respiratory function testing, chest X-ray examination, computed tomography (CT) scan, and peripheral blood analysis. All preoperative evaluations were performed routinely within 56 days before surgery. Interlobar fissures were assessed by preoperative chest CT scans and based on the intraoperative findings.

### SLR

**Fig. 1:**
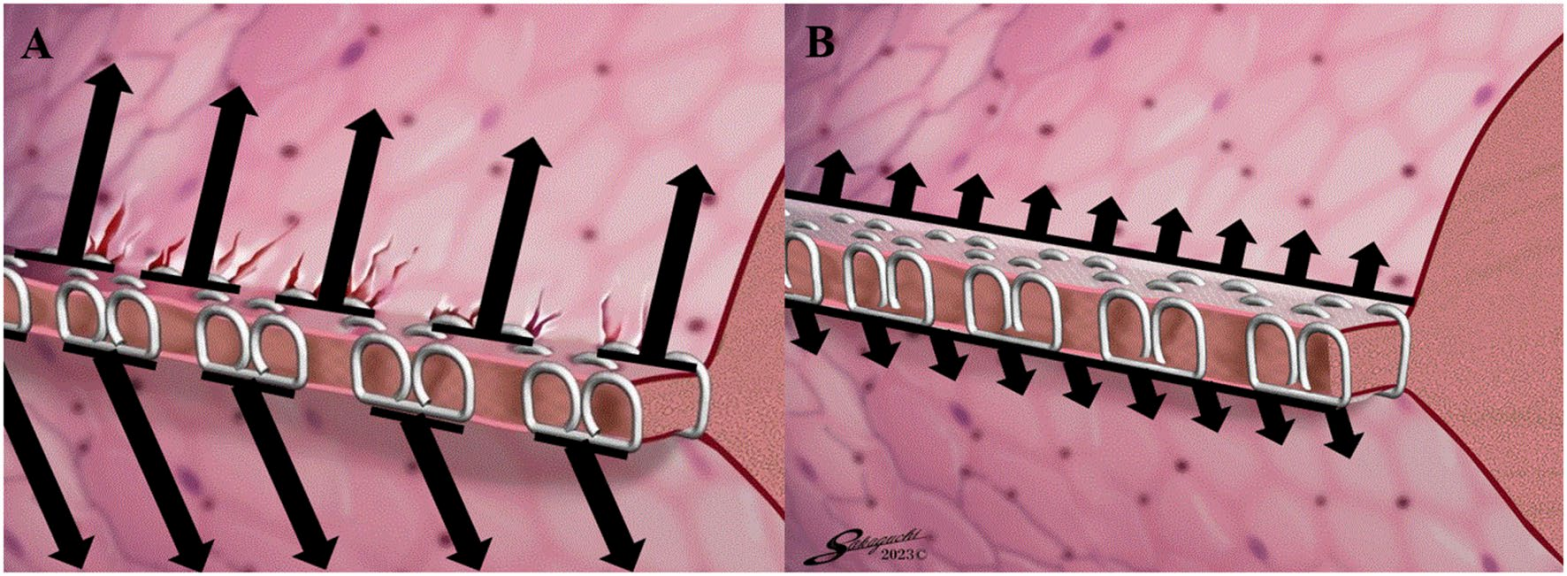
Tension is applied to the needle holes in the staple line at the time of postoperative inflation of the lungs or excessive inflation with coughing. A staple applied without suture-line reinforcement (SLR) causes tension at the point of every needle hole (**A**); however, a staple applied with SLR applies tension to the staple line on the surface evenly and compresses the staple-line softly, resulting in dispersed tension on the needle hole (**B**). Arrow point: tension at the time of lung expansion

The SLR used in this clinical study was the ECHELON ENDOPATH® Staple Line Reinforcement (Ethicon Endo-Surgery Inc., Cincinnati, OH, USA) (Fig. 1). The SLR has a three-layer structure, which includes VICRYL® (polyglactin 910) mesh coated with PDS® (polydioxanone) on both sides, attached to conventional staples. The SLR distributes tension on the tissue by converting the point tension on the needle hole into surface tension. Because the lung is fragile tissue, tissue protection is expected not only at the time of suturing, but also during lung expansion postoperatively. This study utilized the combination of an ENDPATH® STAPLER Powered ECHELON FLEX® + GST System Plus Long Articulating Endoscopic Liner Cutter compatible cartridges (gold 1.8 mm, green 2.0 mm, and black 2.3 mm) (Ethicon Endo-Surgery Inc., Cincinnati, OH, USA), in addition to the bioabsorbable SLR.

### Procedure

For patients who met the selection criteria, an automatic suture device with a combination of cartridges and SLR were used for interlobular fissures. After the removal of the pulmonary lobe, a water sealing test was performed at pressures of 5, 10, 15, and 20 cmH_2_O. The presence of air leakage from the staple line at each pressure and air leakage location were recorded. The air leakage sites were defined as follows: at the edges of the staple line, the side of the staple line, and the staple line junction. If air leakage occurred during evaluation, repair was performed. This study used a conventional three-bottle suction system to evaluate air leakage and the chest drainage tube was removed when there was no air leakage. The duration of persistent air leakage, the time needed for indwelling chest drainage, length of hospital stay, and postoperative complications within 30 days after surgery were all assessed.

### Outcomes

The primary study endpoint was air leakage from the staple line during the intraoperative water sealing test. The secondary endpoints for efficacy were pressure resistance of the SLR, location of the air leakage, and the duration of air leakage after surgery. The secondary endpoints for safety were prolonged air leakage, pulmonary complications, and any other dysfunction of the SLR within 30 days after surgery. Prolonged air leakage was defined as persistent air leakage for > 5 days after surgery or attempted additional treatments, such as pleurodesis or reoperation. Pulmonary complications included atelectasis, respiratory failure, pneumonia, empyema, and bronchopleural fistula.

In addition, the patients were divided into a low (< 70%) and a high (≥ 70%) forced expiratory volume in 1 s (FEV1) % groups according to the results of the preoperative respiratory function test. The primary and efficacy endpoints were compared between the two groups.

### Historical control and sample size estimation

The purpose of this single-arm prospective study was to investigate the efficacy and safety of SLR. However, we also plan to conduct a prospective randomized study comparing SLR and conventional staples in the future. We enrolled 200 consecutive patients who underwent pulmonary resection using conventional staples without SLR at our institution, who met the same selection criteria as in our study, between September, 2019 and February, 2021, and analyzed their data. The ENDPATH® STAPLER Powered ECHELON FLEX® + GST system and Medtronic Signia™ Stapling System (Medtronic plc., Dublin, Ireland) were the conventional staples used in the historical control group. Based on our findings, 183 and 17 patients had primary lung cancer and pulmonary metastases, respectively, 57 (28.5%) of whom had air leakage from the staple line. As an historical control using conventional products without SLR, we estimated the incidence of air leakage between SLR and conventional products based on our own experience at this institution. To conduct an exact test based on the binomial distribution of the percentage of air leakage, using 28.5% as the threshold value and 10% as the expected value for the percentage of air leakage occurrence for SLR, the following results were obtained at a significance level of 2.5% on one side to maintain a statistical power of 80%, and 43 cases were needed. Therefore, the target number of cases was set at 48, to include the expected dropout cases.

### Monitoring

Monitoring was performed to assess whether the study was conducted in accordance with the protocol and the data were appropriately collected. The assigned individual who was not a researcher in the study reviewed the consent form obtained from the participants, medical records, case report form of all participating individuals, recorded videos of the surgeries related to the primary end point and created a monitoring report for each patient.

### Statistical analysis

The demographic characteristics of the patients are expressed as median values with an interquartile range for continuous variables and as the frequency and proportion for categorical variables. The efficacy and safety of SLR in prospective studies were evaluated as follows. The primary analysis was done to calculate the incidence and its 95% confidence interval (CI) for the primary endpoint (air leakage from the staple line) and an exact binomial test with the null hypothesis of 28.5% incidence of air leakage from staple line was performed at a one-sided 2.5% significance level. The secondary endpoints, including the duration of air leakage after surgery, time required for indwelling chest tube drainage, and length of hospital stay were calculated and their median, quartiles, and CI of the median were obtained. As a secondary analysis, the patients were divided into low (< 70%) and high (≥ 70%) FEV1.0% groups according to the respiratory function test and the primary and efficacy endpoints were evaluated between the two groups. When comparing the results of this exploratory study with those of the historical controls, the incidences of air leakage from the staple line and intraoperative air leakage were calculated and the differences were examined using Fisher’s exact test. The median and quartiles for the duration of air leakage after surgery, time required for indwelling chest tube drainage, and length of hospital stay were calculated. The differences in median values were examined using the Wilcoxon rank-sum test. All statistical analyses were conducted with EZR version 1.40 (Saitama, Medical Center, Jichi Medical University, Saitama, Japan), a graphical user interface for R (The R Foundation for Statistical Computing, Vienna, Austria) [7]. A *P* value of < 0.05 was considered significant and multiplicity of tests was not considered. For the secondary endpoints, secondary and exploratory analyses were performed.

## Results

**Fig. 2:**
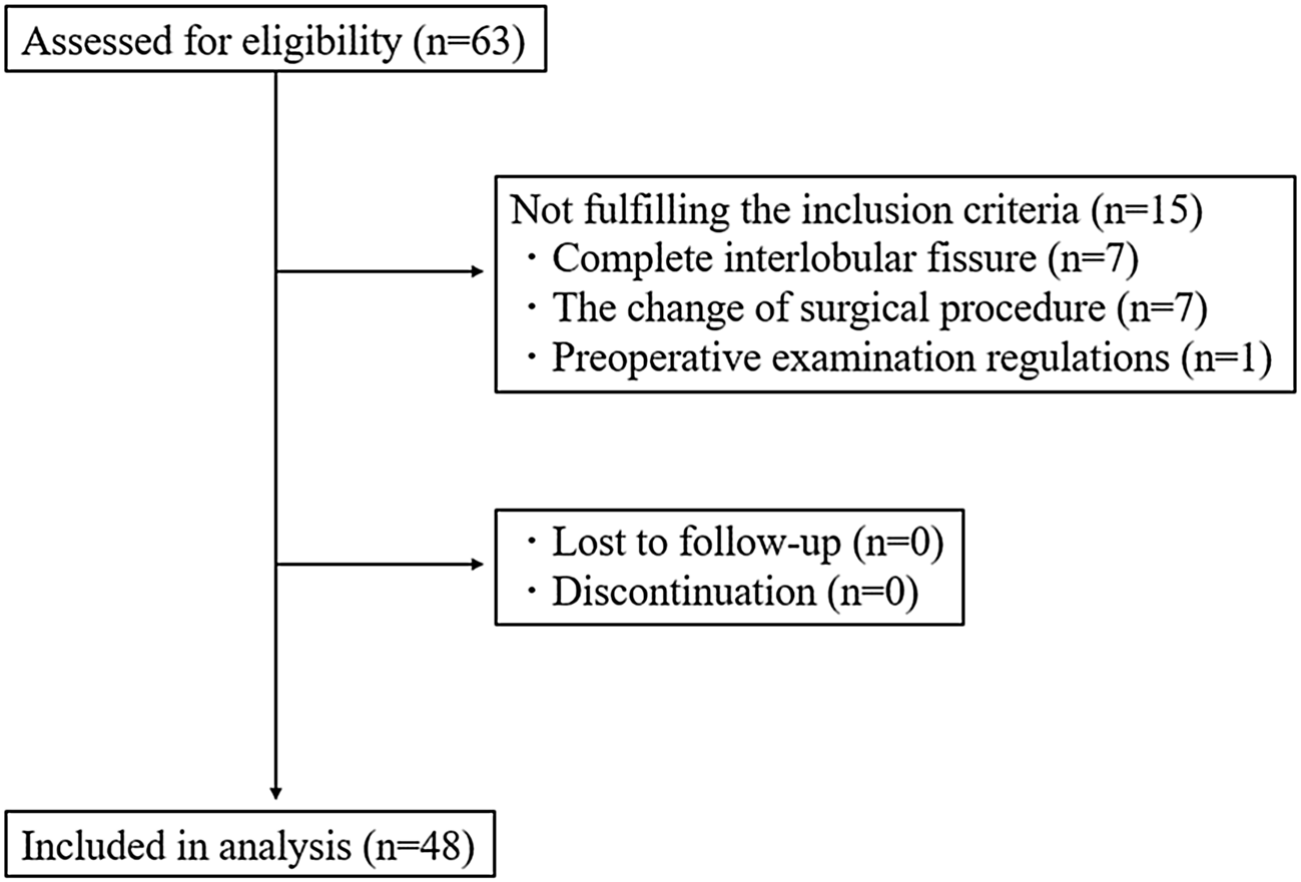
Flowchart of the patients included in this study

The subjects of the final analysis were 48 of the 63 eligible patients, who met the inclusion criteria (Fig. 2). Table 1 summarizes the patients’ characteristics. Fifteen patients were excluded from the analysis for the following reasons: seven had complete interlobar fissures of the lung diagnosed intraoperatively, seven underwent a change in surgical procedure as a result of intraoperative findings, and one deviated from the preoperative examination regulations. There were 22 male and 26 female participants, with a mean age of 69 years. In total, 25 patients presented with a smoking history and 12 had COPD. The number of patients undergoing VATS and RATS was 26 (54.2%) and 22 (45.8%), respectively. There were 41 (85.4%) patients with primary lung cancer, 5 (10.4%) patients with pulmonary metastases, and 1 (2.1%) patient with a benign tumor.

**Table 1:** Characteristics of the patients

Factors	All patients
	*n* = 48
Age	
Median (IQR)	71.5 (64–76.5)
Sex	
Male	22 (45.8)
Female	26 (54.2)
Smoking history	
Yes	25 (52.1)
Pack year	
Median (IQR)	1.25 (0–32.0)
Comorbidities	
Interstitial pneumonia	2 (4.2)
COPD	12 (25)
Diabetes mellitus	5 (10.4)
Preoperative pulmonary function	
VC (L)	2.78 (2.44–3.44)
FEV1.0 (L)	2.13 (1.90–2.48)
FEV1.0%	76.7 (70.5–85.7)
%DLCO	88.2 (81.7–100.0)
Tumor location	
Right upper	19 (39.6)
Right middle	6 (12.5)
Right lower	6(12.5)
Left upper	7 (14.6)
Left lower	10 (20.8)
Surgical procedure	
VATS	26 (54.2)
RATS	22 (45.8)
Operation time (min)	
Median (IQR)	170.5 (145.3–199)
Blood loss (ml)	
Median (IQR)	10 (5–10)
Primary lung cancer	
Adenocarcinoma	33 (68.8)
Squamous cell carcinoma	8 (16.7)
Pulmonary metastases	
Yes	5 (10.4)
Benign tumor	
Yes	2 (4.2)
Tumor size (mm)	
Median (IQR)	27 (15–36.5)

Values are presented as n (%). Continuous variables are presented as the median and interquartile range

*IQR* interquartile range, *COPD* chronic obstructive pulmonary disease, *VC* vital capacity, *FEV1.0* forced expiratory volume in 1 s, *DLCO* carbon monoxide diffusing capacity, *VATS* video-assisted thoracic surgery, *RATS* robot-assisted thoracic surgery

Table 2 shows the intraoperative and postoperative findings. The incidence of intraoperative air leakage was 39.6% (19/48), 6.3% (3/48) of which were from the staple line. The location of air leakage was the side of the staple line at 15 cmH2O in all three patients. Intraoperative air leakage from areas other than the side of the staple line were from the interlobular fissure dissection plane. Soft coagulation and fibrin glue were used as additional management in 27 (56.3%) and 38 (79.2%) patients, respectively. No malfunction of the SLR or stapling device was found. The median duration of air leakage after surgery, time required for indwelling chest drainage, and length of hospital stay were 0, 2, and 10 days, respectively. The only pulmonary complication observed after surgery was prolonged air leakage, which was noted in two patients (4.2%), with one case resolving spontaneously in 7 days and 1 developing air leakage on postoperative day (POD) 3 and requiring reoperation on POD 11. During reoperation, air leakage from a laceration of the remaining lung parenchyma was found but there was no sign of air leakage from the staple line. The chest drainage tube was removed on POD 13. Table 3 presents the 95% CI and results of the comparison of the study findings to those of historical controls for the primary endpoints and the 95% CI for the secondary endpoints. Air leakage from the staple line was significantly less frequent in the study subjects than in the historical controls (*P* = 0.001).

**Table 2:** Intraoperative and postoperative findings

Factors	All patients
	*n* = 48
Interlobular fissure using SLR	
Upper–lower	33
Upper–middle	22
Middle–lower	9
Number of SLRs	
Upper–lower	47
Upper–middle	36
Middle–lower	10
Height of the cartridge	
1.8 mm	20
2.0 mm	69
2.3 mm	8
Intraoperative air leakage	
None	29 (60.4)
Staple line	3 (6.3)
Others	16 (33.3)
Location of air leakage	
First end of the staple line	0
Side of the staple line	3 (6.3)
Later end of the staple line	0
Staple-line junction	0
Others	16 (33.3)
Intraoperative management of air leakage	
Soft coagulation	27 (56.3)
Fibrin glue	38 (79.2)
Suture of lung parenchyma	0
Malfunction of SLR	
Positive	0
Duration of air leakage after surgery (days)	
Median (IQR)	0 (0–0)
Time of indwelling chest drainage (days)	
Median (IQR)	2 (2–3)
Length of hospital stay (days)	
Median (IQR)	10 (9–12)
Pulmonary complications	
Prolonged air leakage	2 (4.2)

Values are presented as n (%). Continuous variables are presented as the median and interquartile range

*IQR* interquartile range, staple-line reinforcement

**Table 3:** Primary and secondary endpoints of this study

Outcomes	Event	Occurrence rate	95%CI	threshold	*P*-value	Significance level
Primary endpoint						
Intraoperative air leakage	19/48	39.6%	25.8–54.7%			
Air-leakage from the staple line	3/48	6.3%	1.3–17.2%	28.5	0.001	One-sided 0.025

Outcomes	*n*	Median (IQR)	95%CI			
Secondary endpoints						
Duration of air leakage after surgery (day)	48	0 (0–0)	-0.002–0.668			
Time of indwelling chest drainage(day)	48	2 (2–3)	2.000–3.000			
Length of hospital stay(day)	48	10 (9–12)	9.772–11.518			

*IQR* interquartile range

The low (< 70%) and high (≥ 70%) FEV1.0% groups comprised 12 (25%) and 36 (75%) patients, respectively. Table 4 shows the intraoperative and postoperative findings according to FEV1.0%. There was no significant difference in the rates of intraoperative air leakage and air leakage from the staple line, duration of air leakage after surgery, time required for indwelling chest tube drainage, and length of hospital stay between the FEV1.0% < 70% and FEV1.0% ≥ 70% groups.

**Table 4:** Intraoperative and postoperative findings according to the forced expiratory volume in 1 s

Outcomes	All patients	FEV1.0% < 70%	FEV1.0% ≥ 70%	*P*-value
	*n* = 48	*n* = 12	*n* = 36	
Intraoperative air leakage				
Positive	19 (39.6)	3 (25)	16 (44.4)	0.316
Negative	29 (60.4)	9 (75)	20 (55.6)	
Air leakage from the staple line				
Positive	3 (6.3)	1 (8.3)	2 (5.6)	1.0
Negative	45 (93.8)	11 (91.7)	34 (94.4)	
Duration of air leakage after surgery (days)				
Median (IQR)	0 (0–0)	0 (0–0)	0 (0–0.5)	0.125
Time of indwelling chest drainage (days)				
Median (IQR)	2 (2–3)	2 (2–3)	2 (2–3.25)	0.621
Length of hospital stay (days)				
Median (IQR)	10 (9–12)	10 (9–12)	10 (9–12)	0.932

Values for categorical variables are presented as n (%) and assessed with the Fisher’s exact test. Variables for continuous variables are expressed as the median and interquartile range and were examined using the Wilcoxon rank-sum test

*FEV1.0* forced expiratory volume in 1 s, *IQR* interquartile range

Supplemental Table 1 summarizes the clinical characteristics of the SLR and historical control groups. The historical control group had significantly more male patients, those with a high smoking pack-year, and those with worse respiratory function than the SLR group. Supplemental Table 2 shows the intraoperative and postoperative findings of the SLR and historical control groups. The SLR group had a significantly lower incidence of air leakage from the staple line and a significantly shorter time of indwelling chest tube drainage and length of hospital stay than the historical control group. Supplemental Table 3 presents the intraoperative and postoperative findings according to gender between the historical control and SLR groups. The male and female patients in the SLR group had a significantly lower incidence of air leakage from the staple line and a significantly shorter length of hospital stay than those in the historical control group.

## Discussion

Air leakage is one of the most frequent complications of pulmonary resection, with an incidence reported to range from 8% to 26% [8]. Various techniques and medical materials have been used to control intraoperative air leakage. A prospective randomized study of patients who underwent pulmonary resection revealed that fibrin glue and fleece-bound sealing reduced the risk of intraoperative and postoperative air leakage significantly [9–11]. Similarly, a previous study on bioabsorbable polyglycolic acid (PGA) sheets found that they were effective in reducing the duration of prolonged air leakage after pulmonary resection [12]. These medical materials are used for intraoperatively damaged pulmonary areas other than the staple lines. Conversely, buttressed staples have recently emerged as a better alternative to staples themselves. Bioabsorbable buttressed staples have been applied in various organs, including the lungs, to provide additional support to the resection line by overlaying the reinforcement on the staple line [13–15].

A previous retrospective study on the efficacy of PGA buttressed staplers showed that the incidence of postoperative air leakage from the staple line for dividing incomplete interlobular fissures was significantly lower when PGA buttressed staplers (9.6%) were used than when non-buttressed staplers (22.4%) were used [16]. Another study found that the rate of postoperative air leakage after pulmonary resection including wedge resection was 7% in patients with buttressed staplers [17]. We consider that the advantage of the PGA buttress lies in the fact that it directly reinforces around the staple hole and inhibits air leakage from the needle hole. Our results showed that the incidence of intraoperative air leakage from the staple line and from interlobular fissure dissection plane was 6.3% (3/48) and 33.3% (16/48), respectively, and that of postoperative prolonged air leakage was 4.2% (2/48). The occurrence of intraoperative air leakage in this study was generally acceptable compared with that reported in previous studies.

As shown in Fig. 1, a nonbuttressed staple maintains compression of the lung parenchyma in the form of a band with a collection of points by the staple needle penetrating the tissue, which may cause tension at the needle hole and air leakage around the staple line. This SLR has a three-layer structure, which is a polyglactin 910 mesh coated with polydioxanone on both sides. A coating of polydioxanone covers the coarse mesh of the polyglactin 910, making the SLR a soft but tight sheet. Therefore, it applies tension to the staple line on the surface evenly and compresses it softly, resulting in less tension on the needle hole and resulting in less intraoperative air leakage. Long term, SLR takes 17 weeks to be absorbed, which is shorter than for PGA buttresses. However, SLR shows a significantly greater staple pull-through force within 14 days in an in vitro solution [18]. Moreover, both polyglactin 910 and polydioxanone, which are SLR materials, are frequently used for suturing wounds and known to have no adverse effects on wound healing [19, 20]. SLR did not adversely affect wound healing and the compressed tissue was partially replaced and infiltrated by fibrous tissues. Furthermore, the staple line on the pleural aspect was covered by a thin fibrous layer within 120 days of in vivo examination [18]. Therefore, SLR effectively reduces pressure on the staple line for a sufficient period during a sudden increase in peak airway pressures, including coughing or strenuous activity. In fact, there were no cases of lung collapse after chest drain removal in our study.

A previous single-arm prospective study focusing on the efficiency of variable-height staple technology in pulmonary resection described a trend toward a lower incidence of air leakage with the use of a cartridge intended for thick tissues [21]. During our study period, we used a higher height staple, considering a 0.2 mm thickness of SLR. In all three cases of intraoperative air leakage from the staple line, the 2.0-mm height staple was used for pulmonary resection. However, in two of the three cases, air leakage was confirmed from the staple hole using SLR in relatively thin lung tissues without emphysema. We think that the staple height was too high for the tissues used in those cases.

In the present study, we used SLR in all patients who underwent lobectomy during the study period, regardless of lung frangibility, because there were no previous data on the effectiveness of SLR. When compared with the historical control group, not only were the intraoperative outcomes better, but also the time required for indwelling chest tube drainage and length of hospital stay were shortened. The benefits of using this material may also include reducing medical costs. Because SLR is a novel medical material, it is necessary to identify which patients would benefit more from the application of SLR. In a study comparing buttressed and nonbuttressed staple lines in lung volume reduction surgery for severe emphysema, the incidence of prolonged air leakage was significantly lower when buttressed staples were used [22]. A study using the Japanese Nationwide Database on lobectomy for primary lung cancer found that a preoperative cumulative smoking dose is a risk factor for pulmonary complications, including prolonged air leakage [23]. We considered COPD and smokers to be good candidates for SLR based on the results of the above studies, and we compared the efficacy of SLR based on the FEV of 1.0% in this study, but found no significant difference in the intraoperative and postoperative outcomes between the FEV1.0% < 70% and FEV1.0% ≥ 70% groups. This indicated that SLR was more effective for patients with low pulmonary function. Further case accumulation, especially of patients with low pulmonary function is required.

The current study had some limitations. First, it was conducted at a single center and the sample size was small, although the statistical power was maintained. Second, a historical control group was compared with the SLR group. Although the inclusion of historical controls is preferred based on the previous literature, there were no data on intraoperative air leakage from the staple line without SLR in the previous studies. In a similar report, the incidence of postoperative air leakage after dividing incomplete interlobular fissures with nonbuttressed staplers was 22.4% [16]. This value is within the expected proportion of intraoperative air leakage from the staple line without SLR in our historical control group. Moreover, the historical control and SLR groups differed in terms of some characteristics. In accordance with the study protocol, consecutive eligible cases were collected. However, the historical control group had a significantly higher proportion of male patients, those with a high smoking pack-year, and those with a lower respiratory function than the SLR group. The inclusion criteria for the SLR and historical control groups were similar and differences between these groups can be related to the fact that there were more male patients with poor pulmonary function who underwent sublobar resection and many could not undergo surgery because of advanced-stage disease during the study period. In this study, there were only three cases of air leakage from the staple line, which is the primary outcome. Hence, it was challenging to perform multivariate analysis. To address this issue, Supplemental Table 3 examined the association between the data in this study and the historical control groups by gender. Similar to the results shown in Supplement Table 2, air leakage from the staple line and the length of hospital stay differed significantly regardless of sex. In the future, we plan to conduct a larger prospective randomized study to compare SLR with the conventional product. Finally, sublobar resection was not included in this study. The efficacy of SLR for wedge resection or segmental resection needs to be studied separately.

In conclusion, the incidences of air leakage from the staple line and prolonged air leakage were 6.3% and 4.2%, respectively, in patients who underwent pulmonary lobectomy with SLR, demonstrating its efficiency. Moreover, the safety of using SLR was demonstrated by the fact that there was no malfunction in the SLR or stapling device. The findings of this pilot study provides some insight into the planning of future trials comparing SLR with conventional products.

### Supplementary Information

Below is the link to the electronic supplementary material.

Supplementary file1 (DOCX 20 KB)

Supplementary file2 (DOCX 17 KB)

Supplementary file3 (DOCX 20 KB)
