# Supplementary material for: Safety and efficacy of new staple-line reinforcement in lung resection: a prospective study of 48 patients
Source: Surg Today. 2024 Feb 21;54(7):779–86. doi: 10.1007/s00595-024-02798-x (PMC11189967; doi:10.1007/s00595-024-02798-x)
Supplement: Supplementary file 1 — Supplementary file1 (DOCX 20 KB) [file 595_2024_2798_MOESM1_ESM.docx]

**Supplemental Table 1. Characteristics of the patients**

| Factors | | SLR group | Historical group | *P*-value |
| --- | --- | --- | --- | --- |
|  |  | n = 48 | n = 200 |  |
| Age | Median (IQR) | 71.5 (64-76.5) | 70 (64-76) | 0.674 |
| Sex | Male | 22 (45.8) | 138 (69) | 0.006 |
|  | Female | 26 (54.2) | 62 (31) |  |
| Smoking history | Yes | 25 (52.1) | 135 (67.5) | 0.063 |
| Pack year | Median (IQR) | 1.25 (0-32.0) | 25 (0-50) | <0.001 |
| Comorbidities | Interstitial pneumonia | 2 (4.2) | 7 (3.5) | 0.687 |
|  | COPD | 12 (25) | 57 (28.5) | 0.721 |
|  | Diabetes mellitus | 5 (10.4) | 22 (11) | 1.0 |
| Preoperative pulmonary function | VC (L) | 2.78 (2.44-3.44) | 3.18 (2.69-3.9) | 0.011 |
|  | FEV1.0 (L) | 2.13 (1.90-2.48) | 2.24 (1.87-2.73) | 0.255 |
|  | FEV1.0% | 76.7 (70.5-85.7) | 74 (69-79.5) | 0.013 |
|  | %DLCO | 88.2 (81.7-100.0) | 82.3 (68.2-93.7) | 0.018 |
| Tumor location | Right upper | 19 (39.6) | 78 (39) | 0.245 |
|  | Right middle | 6 (12.5) | 16 (8) |  |
|  | Right lower | 6(12.5) | 46 (23) |  |
|  | Left upper | 7 (14.6) | 35 (17.5) |  |
|  | Left lower | 10 (20.8) | 24 (12) |  |
| Surgical procedure | VATS | 26 (54.2) | 104 (52) | 0.873 |
|  | RATS | 22 (45.8) | 96 (48) |  |
| Operation Time (min) | Median (IQR) | 170.5 (145.3-199) | 180 (156.5-210) | 0.072 |
| Blood loss (ml) | Median (IQR) | 10 (5-10) | 10 (10-30) | <0.001 |
| Primary Lung Cancer | Adenocarcinoma | 33 (68.8) | 139 (69.5) | 0.075 |
|  | Squamous cell carcinoma | 8 (16.7) | 34 (17) |  |
|  | Others | 0 | 13 (6.5) |  |
| Pulmonary metastases | Yes | 5 (10.4) | 13 (6.5) |  |
| Benign tumor | Yes | 2 (4.2) | 1 (0.5) |  |
| Tumor size (mm) | Median (IQR) | 27 (15-36.5) | 23 (17-32) | 0.547 |

Values for categorical variables are presented as n (%) and assessed with the Fisher’s exact test. Variables for continuous variables are expressed as median and interquartile range and were examined using the Wilcoxon rank-sum test. IQR, interquartile range; COPD, chronic obstructive pulmonary disease.; VC, vital capacity; FEV1.0, forced expiratory volume in 1 second; DLCO, carbon monoxide diffusing capacity; VATS, video-assisted thoracic surgery; RATS, robot-assisted thoracic surgery
