# Supplementary material for: Safety and efficacy of new staple-line reinforcement in lung resection: a prospective study of 48 patients
Source: Surg Today. 2024 Feb 21;54(7):779–86. doi: 10.1007/s00595-024-02798-x (PMC11189967; doi:10.1007/s00595-024-02798-x)
Supplement: Supplementary file 2 — Supplementary file2 (DOCX 17 KB) [file 595_2024_2798_MOESM2_ESM.docx]

**Supplemental Table 2. Intraoperative and postoperative findings of the study group vs. the historical group**

| Outcomes | | All patients | SLR group | Historical group | *P*-value |
| --- | --- | --- | --- | --- | --- |
|  |  | n=248 | n = 48 | n = 200 |  |
| Intraoperative air leakage | Positive | 90 (36.3) | 19 (39.6) | 71 (35.5) | 0.619 |
|  | Negative | 158 (63.7) | 29 (60.4) | 129 (64.5) |  |
| Air leakage from staple-line | Positive | 60 (24.2) | 3 (6.3) | 57 (28.5) | <0.001 |
|  | Negative | 188 (75.8) | 45 (93.8) | 143 (71.5) |  |
| Duration of air leakage after surgery (days) | Median (IQR) | 0 (0-0) | 0 (0-0) | 0 (0-0) | 0.078 |
| Time of indwelling chest drainage (days) | Median (IQR) | 2 (2-4) | 2 (2-3) | 2.5 (2-4.25) | 0.049 |
| Length of hospital stay (days) | Median (IQR) | 12 (10-15) | 10 (9-12) | 12 (11-15.25) | <0.001 |

Values for categorical variables are presented as n (%) and assessed with the Fisher’s exact test. Variables for continuous variables are expressed as the median and interquartile range and were examined using the Wilcoxon rank-sum test. IQR, interquartile range
