# Supplementary material for: Safety and efficacy of new staple-line reinforcement in lung resection: a prospective study of 48 patients
Source: Surg Today. 2024 Feb 21;54(7):779–86. doi: 10.1007/s00595-024-02798-x (PMC11189967; doi:10.1007/s00595-024-02798-x)
Supplement: Supplementary file 3 — Supplementary file3 (DOCX 20 KB) [file 595_2024_2798_MOESM3_ESM.docx]

**Supplemental Table 3. Intraoperative and postoperative findings by gender of the study group vs. the historical group**

Male

| Outcomes | | All patients | SLR group | Historical group | *P*-value |
| --- | --- | --- | --- | --- | --- |
|  |  | n=160 | n = 22 | n = 138 |  |
| Intraoperative air leakage | Positive | 61 (38.1) | 8 (36.4) | 53 (38.4) | 1.000 |
|  | Negative | 99 (61.9) | 14 (63.6) | 85 (61.6) |  |
| Air leakage from staple-line | Positive | 44 (27.5) | 2 (9.1) | 42 (30.4) | 0.041 |
|  | Negative | 116 (72.5) | 20 (90.9) | 96 (69.6) |  |
| Duration of air leakage after surgery (days) | Median (IQR) | 0 (0-0) | 0 (0-0) | 0 (0-0) | 0.563 |
| Time of indwelling chest drainage (days) | Median (IQR) | 3 (2-5) | 2 (2-4.75) | 3 (2-5) | 0.353 |
| Length of hospital stay (days) | Median (IQR) | 12 (11-15.25) | 10.5 (9-12.75) | 12.0 (11-16) | <0.001 |

Female

| Outcomes | | All patients | SLR group | Historical group | *P*-value |
| --- | --- | --- | --- | --- | --- |
|  |  | n=88 | n = 26 | n = 62 |  |
| Intraoperative air leakage | Positive | 29 (33.0) | 11 (42.3) | 18 (29.0) | 0.320 |
|  | Negative | 59 (67.0) | 15 (57.7) | 44 (71.0) |  |
| Air leakage from staple-line | Positive | 16 (18.2) | 1 (3.8) | 15 (24.2) | 0.032 |
|  | Negative | 72 (81.8) | 25 (96.2) | 47 (75.8) |  |
| Duration of air leakage after surgery (days) | Median (IQR) | 0 (0-0) | 0 (0-0) | 0 (0-0) | 0.111 |
| Time of indwelling chest drainage (days) | Median (IQR) | 2 (2-3) | 2 (2-2.75) | 2 (2-3) | 0.427 |
| Length of hospital stay (days) | Median (IQR) | 11 (10-13) | 10 (9-11) | 12 (10.25-14.5) | <0.001 |

Values for categorical variables are presented as n (%) and assessed with the Fisher’s exact test. Variables for continuous variables are expressed as the median and interquartile range and were examined using the Wilcoxon rank-sum test. IQR, interquartile
